# Infectious medical waste (IMW) and disease transmission: A critical public health challenge in Africa and the global implications – A systematic review

**DOI:** 10.1177/22799036261470478

**Published:** 2026-07-17

**Authors:** Jeffrey N. T. Squire, Michaelann George, Zakirah Allain, Oghenowede Eyawo

**Affiliations:** 1 Department of Social Science, York University, Toronto, Canada; 2 Faculty of Applied Health and Community Studies, Sheridan College, Toronto, Canada; 3 Department of Anthropology, York University, Toronto, Canada; 4 School of Global Health, Faculty of Health, York University, Toronto, Canada

**Keywords:** infectious medical waste, risks, public health, infectious diseases, Africa, global health

## Abstract

**Background:**

Infectious Medical Waste (IMW) from both human and animal healthcare sources have the potential to transmit diseases in human populations due to its pathogenic composition. Conversely, a paucity of data regarding cause-and-effect relationships inhibits our understanding of the extent to which disease transmission occurs. In parts of Africa where the management of IMW is not prioritized, the risks to public health, along with its global implications cannot be ignored.

**Methodology:**

A systematic literature search following the PRISMA guidelines was conducted across five electronic databases including PubMed, Embase, Scopus, ProQuest and Ovid. Relevant studies were identified using keywords such as ‘infectious medical waste’, ‘infectious diseases’, ‘routes of infection’, ‘route of transmission’, ‘epidemics’, ‘diseases’, ‘sub-Sahara Africa’, and ‘infectious waste management’.

**Results:**

Overall, 6721 articles were retrieved from the search, 6634 of which were excluded due to a lack of alignment with the study objectives. After screening, 87 studies were deemed eligible and were included in the review. Our findings identified 11 diseases that are potentially transmissible via IMW. These include Anthrax, Crimean-congo haemorrhagic Fever, Ebola Virus Disease, Hepatitis B, Hepatitis C, Human Immunodeficiency Virus, Lassa Fever, Measles, Monkeypox, Plague, and Rift Valley Fever. Disease transmission may occur via human contact with different classes of IMW, contaminated materials and accessories.

**Conclusions:**

Medical waste can be a source of infectious disease transmission. However, evidence of such a pathway is currently unavailable due to a paucity of scientific data. Therefore, further research is needed to establish cause-and-effect relationships.

## Introduction

Disease transmission and spread in human populations have been studied extensively with research emphasizing factors such as urbanization,^1–3^ fragile healthcare systems,^4,5^ conflict,^6,7^ globalization,^8,9^ climate change^10^ and microbial adaptation^11–13^ as primary drivers. Within this equation, infectious medical waste (IMW) from both human and animal clinical sources remains a largely understudied subject, despite the health risks due to its pathogenic composition. IMW (see Table 1) include discarded human and animal anatomical parts, blood, body fluids, syringes, needles, scalpels, knives and blades (14, 15 16). It also contains clinically contaminated materials and accessories such as clothing, beddings, towels, linens, napkins and facemasks.^14,15^ Despite the risks, IMW management is often poorly prioritized in many parts of Africa. Studies have shown that in most countries, access to vital treatment technologies, transportation systems and disposal facilities is practically inadequate or virtually non-existent.^16–22^ Such haphazard conditions elevate risks of infections in human populations, further compounding existing public health challenges.

**Table 1.** Types, composition and main sources of IMW.

Type	Composition	Main generating sources
Human/animal anatomical waste	Tissues	Operating theatres and surgical wards
	Organs	
	Body parts (excluding teeth, hair and nails)	
	Carcasses	Veterinary Clinics/hospitals
	Bedding	
	Items or body fluids contaminated with blood	
Laboratory waste	Lab/microbial cultures	Laboratories
	Live/attenuated vaccines	
	Human or animal cell cultures	
	Specimens of microorganisms	
	Body fluids of patients	
Human blood/body fluid	Human blood	Medical wards/laboratories
	Body fluid	Maternity Homes
	Blood products	Mortuary and autopsy centres
	Body fluids contaminated with blood	Traditional birth attendants
	Used absorbent material saturated with blood, body fluids or excretions contaminated with blood. E.g., bandages, gauze and sponges	Underground abortion clinics
		Traditional circumcision experts
		Traditional healing centres
Sharps	Needles/syringes/blades/knives/scalpels	Physician’s offices
		Home healthcare
		Psychiatric wards
	Glass capable of causing cuts or punctures	Hospitals, clinics,
		(Poly)clinics
		Family Planning Units

Africa has a longstanding history of infectious diseases outbreaks with major global implications. Within this present decade, 5 public health emergencies of international concern (PHEIC) originating out of the continent has been issued by the World Health Organization (WHO) in line with International Health Regulations (IHR).^23^ These include: the Poliomyelitis epidemic (2014-present), the West African Ebola Virus Disease outbreak (2013-2016), Kivu/Ituri Ebola virus outbreak (2018-2020), Clade II MPox epidemic (2022-2023), and the Clade I MPox epidemic (2014-present). A PHEIC declaration is issued when an extraordinary event requires a coordinated international response.^23^ Researchers have also noted that in the past two decades, the continent has experienced over 1800 public health emergencies, including new and emerging pathogenic infections.^24–26^ In 2018 alone, the WHO recorded 96 new infections disease outbreaks across 36 countries in Africa with Uganda, the Central African Republic (CAR), the Democratic Republic of Congo (DRC) and Sudan reporting the highest frequency events.^25^ Although there is no data linking these outbreaks to IMW, risks cannot be ignored due to the etiology of some of these diseases.

In this study we identify and examine 11 diseases that can potentially be transmitted by IMW. These include Anthrax, Crimean-Congo haemorrhagic fever (CCHF), Ebola Virus Disease (EVD), Hepatitis B (HBV), Hepatitis C (HCV), Human Immunodeficiency Virus (HIV), Lassa Fever, Measles, MPox, the Plague and Rift Valley Fever Virus (RVFV). Through our findings, we aim to inform disease prevention efforts, guide resource allocation and support policy formulation, implementation and monitoring. The findings further seek to strengthen public health surveillance, interventions and responses to threats posed by existing and emerging infectious diseases. By deepening our understanding of the critical yet often overlooked role of IMW in global health security, this study aims to provide public health stakeholders and policy makers across governments, healthcare systems and global health organizations with the insights necessary to effectively address these challenges. Essentially our findings contribute to the ongoing global discourse on improving health systems. The study is among the first of its kind, if not the first to address this subject on such a comprehensive scale. Past some studies have examined healthcare waste and its link to diseases without addressing how specific diseases might be transmitted.^27–29^

## Methods

This systematic review identifies 11 specific diseases capable of being transmitted by IMW with the primary objective of informing public and global health interventions. Whilst the mismanagement of IMW is pervasive across Africa, the study has broader relevance for other developing regions of the world facing similar challenges. Our methodological approach to the study adhered to the Preferred Reporting Items for Systematic Reviews and Meta-Analysis (PRISMA) guidelines^30^ as well as the PRISMA Guidelines for reporting systematic reviews.^31^ This approach was adopted to ensure a rigorous and transparent approach to the review process.

### Literature search

A comprehensive literature search was undertaken over a five-month period from January-May 2025 by 4 researchers (JNTS, MG, ZA and OE). Relevant peer-reviewed studies were extracted in several databases including PubMed, Embase, Scopus, ProQuest and Ovid. The search strategy involved a combination of keywords including ‘risks’ ‘infectious medical waste’; ‘infectious diseases’; ‘routes of infection’; ‘route of transmission’; ‘epidemics’; ‘diseases’; ‘sub-Sahara Africa’; ‘infectious waste management’; ‘anthrax’; ‘Crimean-Congo haemorrhagic fever’; ‘Ebola Virus Disease’; ‘epidemiology’; ‘Hepatitis B’; ‘Hepatitis C’; ‘Human Immunodeficiency Virus’; ‘Lassa Fever’; ‘Measles’; ‘Monkeypox’, and the ‘Plague’. These terms were selected to capture a broad range of studies related to the possible transmission of infectious diseases through IMW and management practices that affect disease spread. A grey literature search was utilized to access data from global sources including the WHO and the African Centres for Disease Control and Prevention (ACDC). The grey literature was instrumental in providing vital data regarding disease outbreaks, types, countries and timescale.

### Inclusion/exclusion criteria

As researchers, we are aware that not all types of waste in healthcare settings can transmit diseases in human populations. Certain types such as chemical, pharmaceutical, genotoxic and radioactive wastes although hazardous, are not infectious and do not pose a direct risk of disease transmission. Therefore, studies relating to the health effects of non-infectious waste from healthcare facilities were excluded from this review. IMW risks to animal populations were also excluded from this review since our focus was on human risks. Further, our review excluded studies published before 2005 to ensure that we stay within a two-decade time frame. Essentially, the studies we excluded were deemed to be out of the scope of our study, have insufficient data and fell outside of our targeted publication period. To ensure relevance and rigor, the inclusion criteria were limited to studies published in English within the last two decades (2005-2025), that highlights the types of diseases that can be transmitted by IMW and their routes of transmission. Studies, regardless of geographical focus were eligible for inclusion if they met these criteria. A combination of academic and grey literature sources including original articles and policy briefs was utilized in the study. No opinion articles were reviewed.

### Study selection, data extraction and aggregation

Three reviewers (JNTS, MG and ZA) initially screened the search results to determine relevance based on the predefined inclusion and exclusion criteria. Full-text articles that met the initial criteria were subsequently retrieved and reviewed independently by the 3 reviewers (JNTS, MG & ZA) to assess their relevance to the research objectives and their potential for inclusion in the study. Any disagreement that arose during the selection process were resolved through discussion between the 3 reviewers (JNTS, MG, and ZA). Where consensus cannot be reached, the fourth reviewer (OE) was consulted to facilitate agreement.

The data extracted from the selected studies were systematically organized and grouped into themes based on each of the diseases addressed in the review, their transmission routes as well as their potential link to IMW. Each disease was qualitatively analyzed to provide insights into its pathogens, transmission routes and outbreaks in Africa since 2005.

## Results

**Figure 1.**
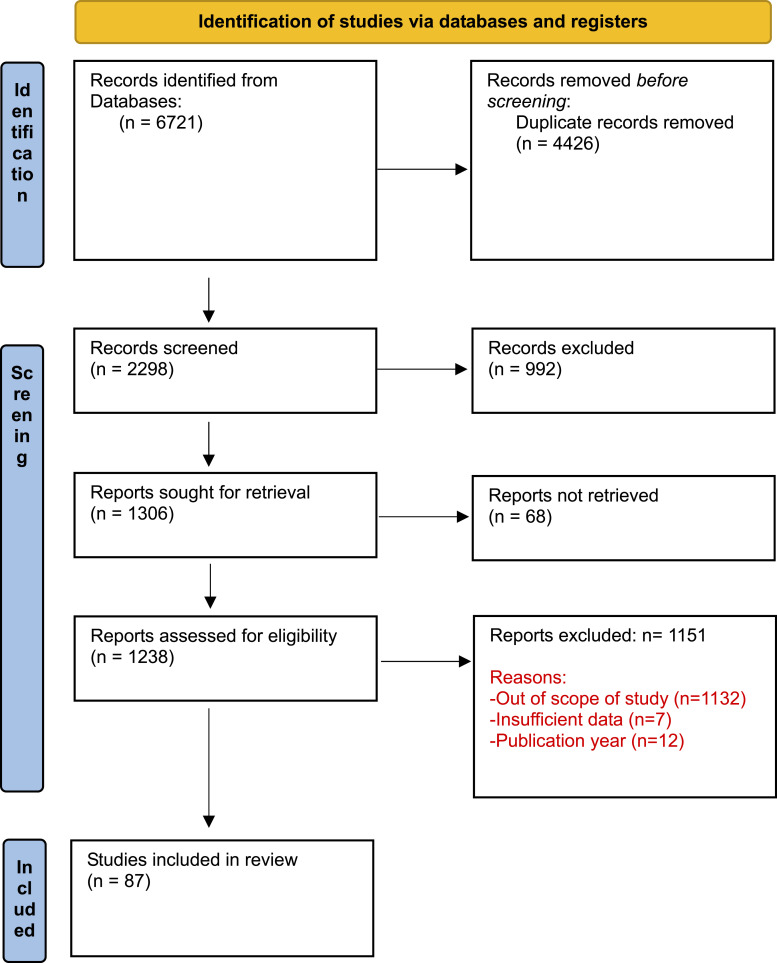
PRISMA Flow Chart.

Overall, 6721 articles were retrieved from the database search out of which 6646 were excluded due to duplication or a lack of alignment with the search objective. After screening, 87 studies were deemed eligible and included in the review. Figure 1 is an illustration of the search results in the PRISMA flow diagram in Figure 1.^30^

## Summary of results

### Anthrax

Although we found no direct evidence of anthrax infection though IMW in Africa, 8 studies suggested a possible transmission route based on the etiology of the disease.^32–40^ Anthrax is a zoonotic disease caused by the bacteria, *Bacillus Anthracis.* Scientists have identified 4 types of the disease; cutaneous, inhalation, gastrointestinal and injection anthrax.^33,35^ In human populations cutaneous anthrax is most common, accounting for 95% of infections globally with a fatality rate of 10-40% when left untreated.^35,37^ Transmission may occur through ingestion, inhalation, handling or injection of spores embedded in the soil, contact with animal byproducts, veterinary waste or contaminated materials.^33,34^ Anthrax spores are scientifically known to be highly resistant and can survive in harsh environmental conditions for prolonged periods, sometimes even decades.^36^ Consequently, the improper disposal of IMW from animal healthcare sources can create conducive conditions for disease transmission. The disease is endemic to Africa where multiple outbreaks have been reported in several countries in recent years.^37–40^

### Crimean-Congo Haemorrhagic Fever (CCHF)

CCHF is a viral disease transmitted to humans primarily through tick-bites or the handling of an infected tick.^41^ Based on our review, 8 studies, found human exposure to viraemic blood, body fluids, and both human and animal anatomical as possible transmission pathways.^42–48^ Additionally, the improper disposal of contaminated medical equipment including sharp objects can be a major risk factor in disease transmission.^43,48^ CCHF is prevalent in Africa and has a case fatality rate of approximately 40%.^41,44,48^ Several African countries including Uganda, Senegal, South Africa and Mauritania have reported outbreaks in recent years.^41,48^ We did not find any data pertaining to a direct link between IMW and CCHF transmission.

### Ebola Virus Disease (EVD)

Based on its etiology and pathogenesis, we found 12 studies that indicates a potential transmission route between IMW and the Ebola virus.^49–60^ This may occur through exposure to infected blood, body fluids as well as contaminated materials such as bedding, towels, linens and clothing of infected individuals.^49–60^ The Ebola virus was first discovered in the Democratic Republic Congo (DRC) during the 1970s and has since become endemic to Africa where multiple outbreaks have been recorded since its discovery.^52,53^ As illustrated in Table 2, more than 18,000 cases of the Ebola virus resulting in over 13,000 fatalities were reported in 7 African countries between 2008 to 2019.^41^ As well, 347 Ebola cases culminating in 192 fatalities in Africa were reported from 2020-2025.^53^

**Table 2.** Outbreaks of EVD in Africa: 2008-2019.

Year	Countries	Cases	Fatalities
2019	Uganda	3	2
2019	DRC	2148	1488
2018	DRC	120	78
2017	DRC	54	33
2014-2016	Guinea, Liberia Sierra Leone	15898	11308
2014	Nigeria, Senegal, DRC	87	57
2012	Uganda, DRC	88	50
2011	Uganda	1	1
2008	DRC	32	14

### Hepatitis B (HBV)

Exposure to infected blood, body fluids, contaminated needles, syringes and sharp objects embedded in the medical waste stream can potentially transmit the hepatitis B virus in human populations.^61^ We did find a possible link between HBV infection in medical waste collectors in 4 studies out of Ethiopia^62–65^ and 1 study out of Libya.^66^ Serologically, the virus is diagnosed though the presence of hepatitis B surface antibody (HBsAb), hepatitis B pre-core antigen (HBeAg), hepatitis B pre-core antibody (HBeAb), hepatitis B surface antigen (HBsAg), or hepatitis B core antibody (HBcAb) sero-marker reactivity.^61,65^

### Hepatitis C (HCV)

HCV is a blood-borne virus which can be transmitted through exposure to needles, infusion bags, syringes and medical equipment.^67^ Two studies undertaken in Ethiopia^64,65^ and one out of Libya^66^ suggested a correlation between exposure to IMW in healthcare settings and HCV infections. This may occur through exposure to contaminated syringes, infusion bags, medical equipment and accessories.^68–70^ Although research shows that large and/or repeated direct percutaneous exposures to blood such as, transfusion, transplantation from infectious donors, and intravenous drug use is the primary mode of transmission,^71^ the risks posed by medical waste cannot be discounted (Table 3).

**Table 3.** Outbreaks of plague in Africa – 2014 2017.

Year	Countries	Cases
2017	Madagascar	2348
2016	Madagascar	62
2015	Madagascar	14
2014	Madagascar	119

### Human Immunodeficiency Virus (HIV)

Percutaneous inoculation from unsterilized sharps and syringes was identified as **major risk factors** for HIV infection through IMW in 8 of our studies.^72–78^ We did not find a direct correlation between HIV infection and exposure to medical waste. Yet, the indiscriminate disposal sharps, blood, syringes and blood products in unsanitary landfills can expose populations, particularly waste pickers to heightened risks.^79–84^ Researchers have noted that although the minimal infectious dose of HIV remains unknown, transmission can be influenced by the volume of blood remaining in a discarded syringe, amount of virus and the depth of the needlestick.^73^

### Lassa fever

Lassa fever is caused by the Lassa virus, which belongs to the family *Arenaviridae.*^85^ The virus is primarily transmitted to humans through the urine of infected rodents.^85^ Our review found that in human populations the virus can be transmitted through direct contact with blood, urine, faeces or other bodily secretions of an infected person.^85–93^ As well, exposure to discarded blood-soaked bandages, linens and other contaminated items in the IMW stream can cause transmission of the virus.^86,88^

### Measles

Measles virus (MV) is a highly infectious disease that belongs to the prototype member of the genus *Morbillivirus,* the subfamily *Paramyxovirinae* and the family *Paramyxoviridae*.^94,95^ The virus spreads primarily through the air when an infected person coughs, talks or sneezes.^96^ Based on its etiology, we found 2 studies that suggested possible transmission through the unsafe handling of contaminated bedding, clothing, wipes, tissues and napkins.^94,95^ Although this avenue presents a low transmission risk, it still cannot be discounted. Despite the availability of vaccines, measles is still one of the leading causes of death among children especially in least developed countries.^95^

### MPox

MPox is a contagious disease transmitted by the monkeypox virus which is part of the same family of viruses as variola, vaccinia, and cowpox.^97^ Transmission typically occurs when the virus enters the host through direct exposure to skin or mucosal surfaces, including oral, anogenital, or ocular tissues.^98–101^ Although we did not find any direct evidence of MPox transmission through IMW, 11 of the studies we reviewed suggested possible infection through contact with infected body fluids, contaminated bedding, clothing and accessories.^96–106^

### The plague

The Plague is a zoonotic disease caused by the bacteria Yersinia pestis, that has its main reservoir in small mammals such as rodents and fleas with a case-fatality ratio of 30% to 60%.^107^ Patients infected with the pneumonic plague can generate respiratory droplets from bloody sputum, urine, feces, sweat, or bubo pus.^108^ The improper disposal of materials used to clean these droplets may lead to human exposure and subsequently, infections. As well, human contact with infected carcasses in veterinary waste may contribute to infections. Although we found no evidence of direct human transmission via IMW, 3 publications highlight risk factors that may include contact infected blood, body fluids, human and animal anatomical waste.^107–109^ The DRC and Madagascar are the two most endemic countries in SSA with the latter recording yearly outbreaks from 2014-2017.^108,109^ As illustrated in Table 4 below, 2543 cases of the Plague were reported from 2014-2017 in Madagascar alone.^41^

**Table 4.** Outbreaks of RVF in Africa.

Year	Countries	Cases
2018	Kenya	94
​	Uganda	4
2016	Niger	348
2012	Mauritania	36
2010	South Africa	237
2010	South Africa	237
2009	Madagascar	236
2008	Madagascar	476

### Rift Valley Fever (RVF)

RVF is zoonotic disease caused by Rift Valley fever virus (RVFV) belonging to family *Bunyaviridae*, genus *Phlebovirus*^110–113^*.* We found no direct evidence of human infection through IMW, although 3 studies highlighted contact with infected blood, organs and animal anatomic waste from veterinary sources as possible transmission pathways.^110–113^ The disease was first identified in Kenya in the 1930s and is now endemic to several African countries including Kenya, Niger, Mauritania, Uganda, South Africa and Madagascar where recent outbreaks have been reported (see Table 4). In a 10-year period 1668 cases of RVF were reported in 6 African countries between 2008-2018.^41^

## Discussion

Exposure to discarded blood, blood products, anatomical waste, body fluids, sharps, contaminated materials and accessories from both human and animal healthcare sources are potential pathways for the transmission of infectious diseases (see Table 5). Despite the risks, our review did not uncover sufficient data to support direct cause-and-effect relationships. However, we did find an association between working with IMW and prevalence of both HBV and HCV in healthcare settings, although it remains unclear whether transmission occurred via IMW or other pathways.^62–66^ In one of the studies, researchers detected **HBV** infections among randomly selected waste collectors from public health facilities in eastern Ethiopia.^63^ A separate study undertaken in Adiss Ababa found higher **HBV** prevalence rates in municipal waste handlers than non-municipal waste handlers.^62^ A comparative study also undertaken in Ethiopia further found a higher seroprevalence of HBV infection among medical waste handlers compared to domestic waste handlers.^65^ In that particular study, the overall seroprevalence of infections for HCV was 4.3% among medical waste handlers and 0.5% for domestic waste handlers.^65^ Also in Libya, research involving 300 medical waste handlers and 300 non-medical waste handlers uncovered higher prevalence rates of HBV and HCV infections in personnel who handled medical waste.^66^

**Table 5.** Diseases, Pathogens and Potential IMW Transmission Pathways based on Scientific Evidence.

Disease	Pathogen	IMW transmission pathway	Studies
Anthrax	Bacteria	Animal anatomical parts	31-39
		Inhalation of spores Ingestion of spores	
CCHF	Virus	Blood	40-47
		Body fluids	
		Secretions	
EVD	Virus	Blood	48-59
		Body fluids	
		Bedding	
HPV	Virus	Blood	60-71
		Body fluids	
		Sharps	
HCV	Virus	Blood	72-76
		Body fluids Sharps	
HIV	Virus	Blood	77-84
		Sharps	
Lassa Fever	Virus	Blood	85-93
		Tissues	
		Body Fluids	
Measles	Virus	Body Fluids	94, 95
		Bedding	
		Linens	
Monkeypox	Virus	Body Fluids	96-106
		Bedding	
		Towels	
Plague	Bacteria	Body fluids	107-109
		Human/animal anatomical waste, Laboratory waste	
RVF	Virus	Animal Anatomical Waste	109-113
		Blood	
		Fluids & Secretions	
		Infected Animal tissue	

### Global health implications – MPox and EVD

Ebola virus disease and MPox, two highly infectious diseases that originated out of Africa have prompted the WHO to make multiple PHEIC declarations in recent years. The Ebola virus was first declared a PHEIC in August 2014 following an initial outbreak in Guinea, West Africa.^52,53^ The West African Ebola Virus Disease outbreak later spread to Liberia, Sierra Leone, Italy, Mali, Nigeria, Senegal, Spain, the United Kingdom, and the United States of America (USA). During this outbreak, close to 30,000 people were infected, culminating in some 11, 325 fatalities, making it the deadliest of the seven outbreaks since its discovery.^52^ The outbreak was eventually declared over in June 2016. A second PHEIC declaration involving the Ebola virus was issued in July 2019 following an initial outbreak in the DRC which later spread to Uganda. By 2019, the Kivu/Ituri Ebola virus outbreak had affected a total of 3,296 people, resulting in 2,196 (67%) deaths.^52^

Similarly, MPox was twice declared a PHEIC by the WHO in 2023 and 2024. The 2024 declaration was issued following an outbreak of Clade 1 MPXV in the DRC which later spread to neighboring countries.^103^ Since the beginning of MPox monitoring in 2022, over 100 000 confirmed cases of clade I and clade II, including over 200 deaths among confirmed cases, have been reported by more than 120 countries as of, July 2024.^105,109^ As well, over 20 000 MPox cases and over 600 deaths caused by MPXV clade I and clade II have been reported in some Africa Union Member States. These include Benin, Cameroon, Central African Republic, Congo, Democratic Republic of Congo, Egypt, Ghana, Liberia, Morocco, Mozambique, Nigeria, Sudan, and South Africa.^106,109^

Based on what is currently known about the etiologies of both the Ebola virus and MPox, it is safe to assume that the presence of pathogenic microorganisms in IMW can create pathways for disease transmission. This may occur as a result of exposure to blood, body fluids, blood products, bedding and contaminated accessories (see table 5). However, there is currently no scientific evidence to substantiate cause-and-effect relationships, prompting the need for further research.

### Limitations

A study of this calibre would have been further strengthened with a formal a priori sample size calculation to highlight cause-and-effect-relationships regarding human exposure to IMW. This fell outside the purview of this study hence, a limitation. In the absence of this, the study highlighted the multiple pathways through which IMW may transmit diseases in human populations. Therefore, a fair degree of caution is warranted in the interpretation of the findings. In the future however, a formal calculation of a sample size would provide statistical clout and a more robust estimation of cause-and-effect relationships.

## Conclusion

Whilst IMW is a clear problem and there are potential transmission routes of human infections, we found no direct or indirect link. This does not negate risks, just a lack of evidence to substantiate cause-and-effect relationships. In instances where we could not find data to support direct causalities, we highlighted potential pathways for transmission and identified ways in which infections may occur, depending on the etiology of the disease. Based on our findings, disease transmission may occur through human exposure to certain classes of IMW. These include improperly discarded blood-borne pathogens, secretions, body fluids, human/animal anatomical waste, sharps, laboratory waste, contaminated materials and accessories. Consequently, the mechanisms by which populations may be infected include needlestick injuries, inhalation, ingestion and contact with improperly disposed infectious agents. Under such conditions, risks to healthcare personnel, especially those on the frontlines, waste handlers and the community are elevated. Therefore, the safe and proper management of IMW is a public health priority. Understanding the multiplicity of ways through which disease transmission occurs via IMW would aid our understanding of disease prevention and response. The subject remains largely understudied and further research is needed to uncover the depth and extent to which IMW transmits diseases in human populations. This is imperative to global public health research.

## Supplemental material

Supplemental material - Infectious medical waste (IMW) and disease transmission: A critical public health challenge in Africa and the global implications – A systematic review Supplemental material for Infectious medical waste (IMW) and disease transmission: A critical public health challenge in Africa and the global implications – A systematic review by Jeffrey N. T. Squire, PhD, Michaelann George, Zakirah Allain, Oghenowede Eyawo in Journal of Public Health Research.

## Data Availability

All information provided in this article could be shared. All data and materials used in this research are available through medical databases. References have been provided.*
